# Research on the mechanism of Ursolic acid for treating Parkinson's disease by network pharmacology and experimental verification

**DOI:** 10.1016/j.heliyon.2024.e34113

**Published:** 2024-07-08

**Authors:** Ao Sun, Yu-fei Li, Yang Miao, Hong-xia Wang, Lin-lin Zhang

**Affiliations:** Department of Pharmacology, Yancheng First Hospital, Affiliated Hospital of Nanjing University Medical School, The First People's Hospital of Yancheng, Yancheng, 224000, Jiangsu, China

**Keywords:** Parkinson's disease, Ursolic acid, CASP8, Apoptosis, Neuroinflammatory, Network pharmacology

## Abstract

The objective of this study was to investigate the potential targets and mechanisms of UA in the treatment of PD. The efficacy of UA in PD was assessed through network pharmacology, molecular docking, and experimental methods. Common target protein-protein interaction (PPI) networks were constructed and visualized using Cytoscape. As a result, 9 key genes, namely CASP3, IL6, IL1B, PTGS2, CREB1, TNF, MAPK3, JUN, and CASP8, were selected. Molecular docking simulations were performed using Discovery Studio 2019 to validate the correlation between UA and the core targets. The results demonstrated a favorable binding affinity between UA and CASP8, IL1B, CASP3, TNF, MAPK3 and IL6. In vivo studies showed UA ameliorated motor dysfunction, and UA can significantly increase the protein expression of tyrosine hydroxylase (TH) in PD mice model. In addition, in vitro experiments confirmed that UA effectively reduced the protein expression of CASP8, CASP3 and MAPK3 in PD cell models and suppressed the gene expression of TNF-α, IL-6, and IL-1β. These findings indicate that the therapeutic effects of UA on PD could be due to its influence on various targets within both the apoptosis and neuroinflammatory signaling pathways. Consequently, this study provides a methodological and theoretical foundation for further elucidating the pharmacological mechanism of UA.

## Graphical abstract

**Figure:**
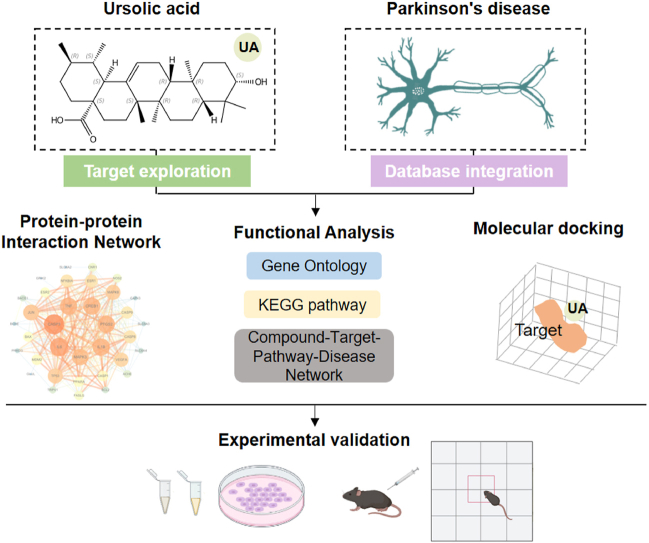

## 1 Introduction

In recent years, there has been a notable rise in the occurrence and fatality rates of Parkinson's disease (PD) as a result of societal and economic advancements leading to an aging population. PD is currently recognized as the second most prevalent neurodegenerative disorder worldwide, with a prevalence exceeding 1.5 % among individuals aged 60 and above [1]. PD does not have a single causative factor but rather arises from a complex interplay of various factors, including environmental exposure, age, and genetics. Advanced age is a significant risk factor for PD, as the body undergoes a continual loss of dopamine neurons with aging. Environmental factors that contribute to PD include exposure to pesticides, lack of physical activity, and head injuries [2].

The primary pathological feature of PD is a significant depletion of dopaminergic neurons in the nigrostriatal region. This depletion is preceded by the accumulation and dissemination of Lewy bodies and neurite inclusion bodies, which primarily consist of misfolded α-synuclein fibers [3]. The abnormal accumulation of α-synuclein is one of the most significant hypotheses explaining the death of neurons in the substantia nigra and striatum in PD. This hypothesis posits that α-synuclein accumulates in the brains of patients and binds to ubiquitin, leading to cellular destruction. These insoluble proteins deposit within the cells, forming Lewy bodies, which ultimately contribute to the development of PD [4].

Recent research has explored various theories regarding the etiology of PD, with particular emphasis on inflammation, microglia, and the inflammasome as potential key contributors to its pathogenesis [5]. Cellular and molecular studies in the human brain have revealed neuroinflammation-related damage in PD patients, with both innate and adaptive immune responses contributing to the progression of the disease [6]. Additionally, oxidative stress leads to cellular toxicity, resulting in neuronal death and accelerating PD progression [7]. Apoptosis is also implicated in the death of dopaminergic neurons in PD [8]. Mitochondrial dysfunction is closely associated with both of these mechanisms and plays a critical role in the pathogenesis of PD [9]. Recently, the role of gut microbiota in neurological diseases has garnered attention. The signaling pathways between the central nervous system and the enteric nervous system involve metabolites, hormones, the immune system, and afferent nerves, with gut inflammation identified as a contributing factor in the pathogenesis of PD [10]. Despite the existence of symptomatic treatment options, the current lack of effective interventions to impede or halt the progression of PD necessitates urgent investigation into the molecular mechanisms underlying its onset and progression, as well as the exploration of novel therapeutic approaches [11]. Notably, research has demonstrated the potential ameliorative effects of certain plant-derived bioactive compounds, including rutin, berberine, and curcumin, on neurodegenerative disorders [[12], [13], [14]].

Ursolic acid (UA), a natural pentacyclic triterpenoid (PT), is frequently present in numerous natural products, primarily derived from aromatic plants and the flesh and skin of various fresh fruits [15]. PTs have been studied for over two decades due to their significant pharmacological properties and low toxicity [16]. PTs were used to treat several cancers without damaging normal cells through toxicity [17]. UA has demonstrated a diverse array of pharmacological properties, encompassing anti-inflammatory, antioxidant, antitumor, and anti-obesity effects [18,19]. Furthermore, mounting evidence suggests that UA exerts a favorable influence on neuroprotection, with a low risk of toxicity [20,21].

Network pharmacology is an academic discipline that investigates the interconnectedness between small molecule drugs and diseases through a holistic, systematic, and comprehensive approach, thereby elucidating the underlying mechanisms of drug action. This discipline facilitates a deeper comprehension of the intricate associations among small molecules, macromolecules, and diseases, thereby offering novel insights for drug research [22]. At the same time, we applied molecular docking to study the interaction between small molecules and target proteins at the atomic level, revealing the molecular mechanism of drug treatment of diseases [23]. The present study aims to explore the anti-PD potential of UA through the utilization of network pharmacology, molecular docking, and experimental validation.

## 2 Materials and methods

### 2.1 Network pharmacology

#### 2.1.1 Obtaining UA-related structure and targets

The molecular structure file in mol2 format was obtained by using the key word "Ursolic Acid" in the Traditional Chinese Medicine Systems Pharmacology Database and Analysis Platform (TCMSP) (https://tcmsp-e.com/). Then the potential targets of UA were searched in TCMSP and SwissTargetPrediction (http://www.swisstargetprediction.ch/) to obtain the list of proteins and converted into the corresponding gene name through the UniProt database.

#### 2.1.2 Identification of PD-related targets

The GeneCards database (http://www.genecards.org) and the disgenet database (https://www.disgenet.org) were used to collect PD-related genes using "Parkinson's disease" as the search term. The targets with strong correlation were screened according to score, and the data of the two databases were merged. Next, the targets of UA and PD were intersected to obtain a list of target genes for further research.

#### 2.1.3 GO and KEGG enrichment analysis

GO and KEGG enrichment analyses of the target genes listed in the previous section were performed using the DAVID database (https://david.ncifcrf.gov/), and the top 10 or 20 pathways were visualized to explore the related biological processes, cellular components, molecular functions, and signaling pathways.

#### 2.1.4 PPI network analysis and screening of the key targets

The target genes listed in the previous section were imported into the String platform (https://www.string-db.org) for protein‒protein interaction analysis, and a PPI network was visualized in Cytoscape 3.7.1 software. The key target genes in the PPI network were screened by using the Cytoscape2.2 plugin.

#### 2.1.5 Construction of the drug-target-disease-pathway network

The drug-target-disease-pathway network was constructed using Cytoscape 3.7.1 software, with nodes representing drugs, targets, diseases and pathways, and edges representing interactions between nodes. The network elucidates the interactions of UA, PD, targets and pathways.

#### 2.1.6 Molecular docking

The UA mol2 file was downloaded from the TCMSP database, and the macromolecular protein structure file was downloaded from the Protein Data Bank (http://www.rcsb.org). Molecular docking was performed using Discovery Studio 2019 Client software.

### 2.2 2.2Experimental verification

#### 2.2.1 Reagents

Ursolic acid (UA, CAS: 77-52-1, B21403) was purchased from Yuanye Biotechnology (Shanghai, China). N-Methyl-4-phenylpyridinium iodide (MPP^+^) (N137206) and 1-Methyl-4-phenyl-1, 2, 3, 6-tetrahydropyridineHydrochloride (MPTP) (M132847) was purchased from Aladdin (Shanghai, China). DMEM/F-12 (Dulbecco's Modified Eagle Medium/Nutrient Mixture F-12), fetal bovine serum (FBS) and antibiotics (streptomycin and penicillin) were obtained from Gibco BRL (Grand Island, NY, USA). The antibodies used in the current study were anti- Tyrosine Hydroxylase (58844S), anti-Phospho-p44/42 MAPK (Erk1/2) (9101S), anti-p44/42 MAPK (Erk1/2) (4695S), anti-cleaved caspase-3 (9661T) and anti-cleaved caspase-8 (9748T), all from Cell Signaling Technology (Danvers, MA, USA), anti-GAPDH (AF5009) from Beyotime Biotechnology (Shanghai, China).

#### 2.2.2 Experimental design

24 male SPF C57BL/6 mice aged 10–12 weeks were selected and acclimated for one week. They were randomly divided into three groups: the Control group, the Model group, and the UA group, with 8 mice in each group. From day 1 to day 14 of the experiment, mice in the UA group were orally administered UA at a dose of 50 mg/kg once daily, while mice in the other two groups received an equivalent volume of physiological saline via oral gavage. During days 10–14 of the experiment, mice in the Model and UA groups were subcutaneously injected with MPTP at a dose of 30 mg/kg·d^−1^ for five consecutive days to induce a PD mouse model. Mice in the Control group were simultaneously injected with an equivalent volume of physiological saline.

#### 2.2.3 Behavioral studies

##### 2.2.3.1 Open-field test

Exploratory and locomotor activities were assessed through the implementation of the Open-field Test. The mice were transferred from the feeding room to the behavioral laboratory 24 h prior, providing them with the opportunity to acclimate to the new environment and move freely. The open-field apparatus comprised a square arena (50 cm × 50 cm × 40 cm) with the floor partitioned into 25 equally sized squares using grids. The regional distribution indices (activity time, movement distance, and residence time in the central region) of the movement track within 5 min were recorded by a digital camera system and ANY-maze software 7.3 version (Stoelting, Wood Dale, USA). Behavioral changes among groups were compared by analyzing both the total activity distance and the activity time in the central region.

##### 2.2.3.2 Climbing Pole Test

Evaluated the coordination ability of mice through the Climbing Pole Test. Fixed a cork ball, with a diameter of 2.5 cm, at the top of a vertically positioned wooden pole measuring 50 cm in height and 1 cm in diameter. The pole was wrapped in gauze to constitute the apparatus for the Climbing Pole Test. Placed the mouse upside down on the ball. When it climbed to the bottom of the pole with both front paws touching the ground, it was considered one test. After each mouse's test, recorded the time it took to climb to the bottom, repeating the process three times. If the time exceeded 30 s, it was recorded as 30 s, with at least a 20 min interval between each of the two tests for the same mouse. Calculated the average of the three test results for each mouse for statistical analysis.

##### 2.2.3.3 Rotarod test

The Rotarod Test was used to assess the motor coordination and balance abilities of mice. Once the Rotarod device was activated, the rotation speed gradually increased from 4 rpm to 40 rpm within 5 min. Mice were placed on the Panlab Rotarod device (Panlab, USA), and the device was started until the mouse fell off the rod, counted as one test. Each experimental mouse underwent one training session before the formal testing, and the formal testing was conducted three times, with at least a 20 min interval between each training or testing session. The time each mouse stayed on the rotating rod during each formal test was recorded, and the average of the three trial results was considered the final outcome for statistical analysis.

#### 2.2.4 Cell culture

The SH-SY5Y cell line was obtained from Yancheng Medical Research Center Nanjing University Medical School. SH-SY5Y, human neuroblastoma cells, were cultured using DMEM/F-12 with 10 % FBS, 2 mM l-glutamine, and 1 % penicillin/streptomycin in an incubator (37 °C, 5 % CO_2_).

#### 2.2.5 Cell viability assay

Cell viability assays for cytotoxicity were performed using the CCK-8 assay. SH-SY5Y cells were seeded in 96-well plates and incubated overnight. Then, different concentrations (0–100 μM) of UA were added for 48 h. To evaluate cell viability under UA treatment, SH-SY5Y cells were pretreated with UA (5, 10, 20 μM) for 24 h and treated with 3 mM MPP^+^ for 24 h. At the end of the stimulation, CCK-8 solution was added to each well and the plates were incubated at 37 °C for 2 h. The optical density (OD450) was measured by a microplate reader (Thermo Fisher Scientific, Waltham, MA, USA)).

#### 2.2.6 Real-time quantitative PCR (RT-qPCR)

SH-SY5Y cells were treated by RNA-easy Isolation Reagent (Vazyme, Nanjing, China) for total RNA extraction. Then the RNA was reverse transcribed to cDNA using the HiScript III RT SuperMix kit (Vazyme, Nanjing, China). RT-qPCR was performed on QuantStudio 5 real-tme PCR systems (Thermo Fisher Scientific, Waltham, MA, USA) with ChamQ Universal SYBR qPCR Master Mix (Vazyme, Nanjing, China). The primer sequences (shown in Table 1) were synthetized by SYNBIO-tech (Suzhou, China). We measured the relative expression levels of three target genes: IL-1β, TNF-α and IL-6, using β-actin as the internal reference gene. The data was calculated with the 2^−ΔΔCT^ method.

**Table 1:** Primer sequences for RT-qPCR.

Gene name	Source	Forward (5′-3′)	Reverse (5′-3′)
IL-1β	*Homo sapiens*	GGATATGGAGCAACAAGTGG	ATGTACCAGTTGGGGAACTG
TNF-α		CAGAGGGAAGAGTTCCCCAG	CCTTGGTCTGGTAGGAGACG
IL-6		ATGAACTCCTTCTCCACAAGCGC	GAAGAGCCCTCAGGCTGGACTG
β-actin		CTGGGACGACATGGAGAAAA	AAGGAAGGCTGGAAGAGTGC

#### 2.2.7 Western blot analysis

SH-SY5Y cells were lysed in RIPA buffer (Beyotime Biotechnology, Shanghai, China)) for 10 min, and total proteins were collected by centrifugation. Then the protein concentration was determined by a BCA protein assay kit (Meiluncell, Shanghai, China). Protein samples were loaded onto a 10 % SDS-polyacrylamide gel and transferred to PVDF membranes (Merck Millipore, MA, USA). After blocking with TBST containing 5 % skim milk powder for 1 h, the membrane was incubated with specific primary antibodies overnight at 4 °C. The membrane was washed 3 times with TBST and incubated with the corresponding secondary antibody at room temperature for 1 h. After washing with TBST, the strength of the strips was measured by chemiluminescence detection reagent and visualized by a ChemiDoc Touch Imaging System (Bio-Rad, Hercules, CA, USA).

#### 2.2.8 Mathematical and statistical analyses

Data are expressed as the mean ± SD (standard deviation) from three independent experiments. Statistical analyses were carried out using one-way ANOVA for multiple groups. Data were considered significant when P ≤ 0.05 (*), P ≤ 0.01 (**), and P ≤ 0.001 (***). All data were analyzed using GraphPad Prism 8.0 (GraphPad Software, Inc., La Jolla, CA, USA).

## 3 Results

### 3.1 Network pharmacology

#### 3.1.1 Target information of UA and PD

**Fig. 1:**
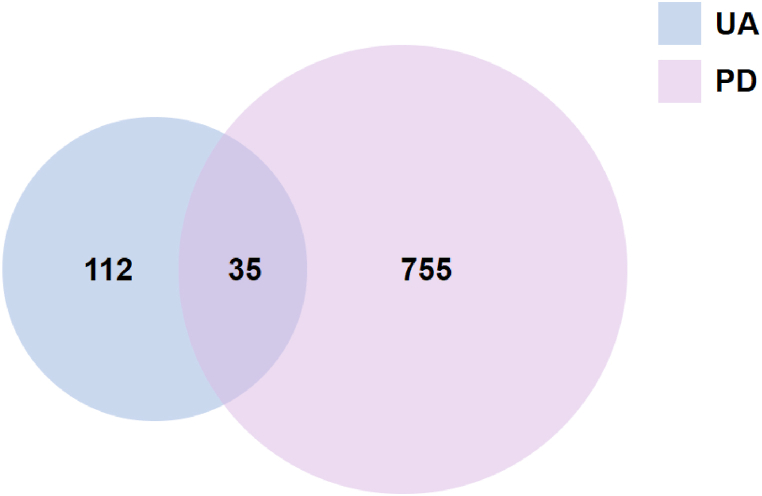
Venn diagram of the target genes of UA and PD.

A total of 147 UA target genes were identified using the TCMSP and SwissTargetPrediction databases. Additionally, 790 target genes associated with PD were collected through the retrieval of GeneCards and disgenet databases. By merging the target genes of UA and PD, a Venn diagram was constructed using Venny2.1 (https://bioinfogp.cnb.csic.es/tools/venny/), revealing 35 overlapping genes (Fig. 1).

#### 3.1.2 GO enrichment and KEGG pathway analyses

**Fig. 2:**
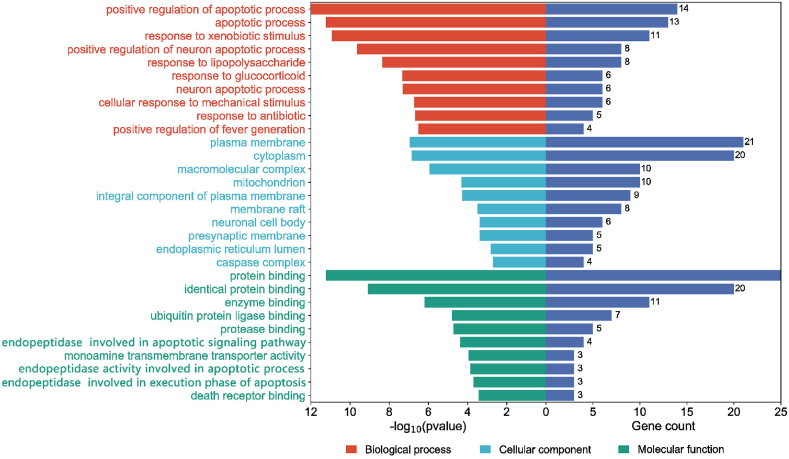
GO enrichment analysis of target proteins.

A bar chart was employed to visually represent the top 10 functions acquired through GO enrichment analysis across different aspects (P < 0.05) (Fig. 2). The most relevant biological processes include positive regulation of apoptotic process, apoptotic process, positive regulation of neuron apoptotic process, and neuron apoptotic process, among others. The most relevant cellular components include plasma membrane, cytoplasm, and macromolecular complex, among others. And protein binding, identical protein binding, and enzyme binding showed strong correlation in molecular function.

**Fig. 3:**
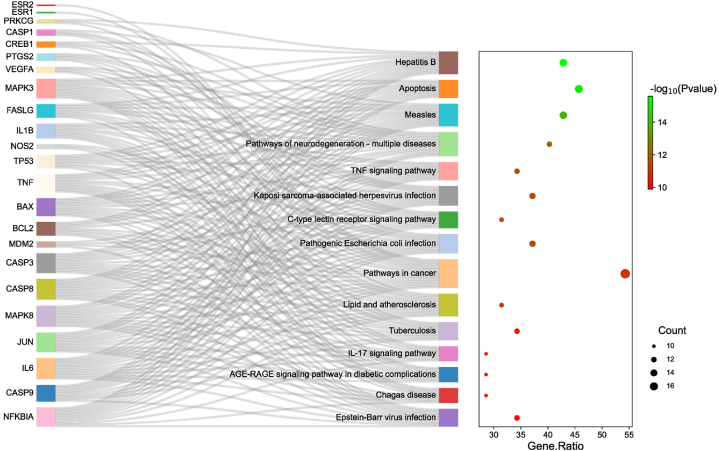
The KEGG pathway analysis of the targets.

The KEGG enrichment analysis revealed that the target genes of UA anti-PD were primarily concentrated in 130 signaling pathways (P < 0.05). The top 15 KEGG pathways were identified based on their P values, and a Sankey＆bubble diagram was generated using R software (Fig. 3). The result showed that Hepatitis B signaling pathway, Apoptosis signaling pathway, Measles signaling pathway, TNF signaling pathway signaling pathway, Pathways in cancer, and Pathways of neurodegeneration-multiple diseases were the significant pathways in the anti-PD effect of UA. Given its close association with PD and its favorable scores in GO and KEGG analyses, we postulated that UA might exert an anti-PD effect via the Apoptosis signaling pathway. Therefore, in the subsequent in vitro experiments, we carried out a preliminary verification of this conjecture.

#### 3.1.3 PPI network analysis and screening of the key targets

**Fig. 4:**
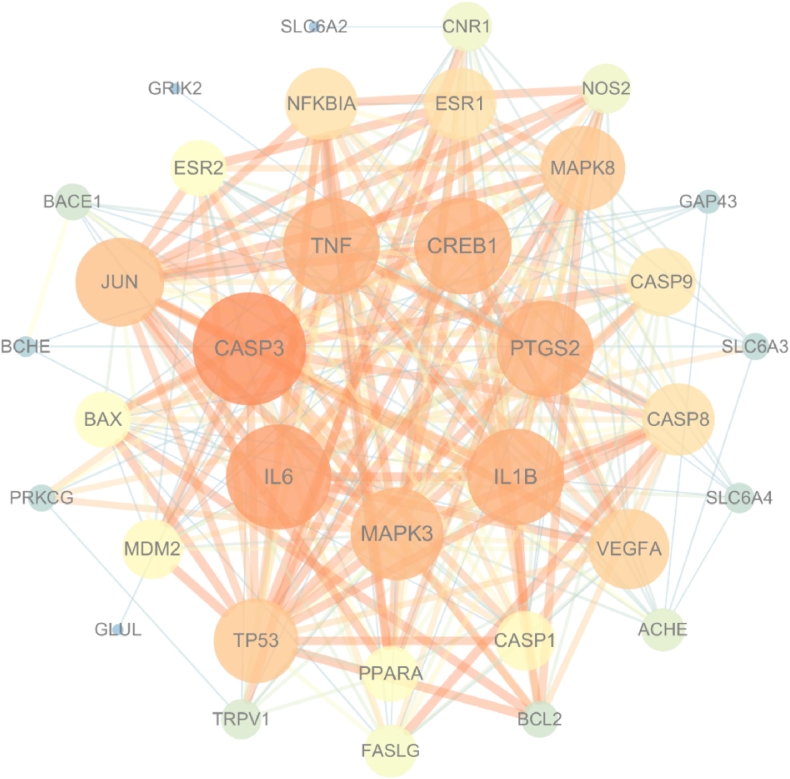
PPI network of potential targets of UA against PD.

The String platform was employed to construct the PPI network of potential UA anti-PD targets (Fig. 4). In this figure, the degree value is correlated with the darkness of the circle color. Similarly, a higher closeness value is indicated by a larger circle area. Moreover, thicker connected lines are indicative of a higher closeness value. The Cytoscape2.2 plug-in within the Cytoscape 3.7.1 software was utilized to identify the key target genes. Based on the plug-in's calculations, 9 key target genes were identified: CASP3, IL6, IL1B, PTGS2, CREB1, TNF, MAPK3, JUN, and CASP8, with degree value > 15.14, betweenness value > 22.11 and closeness value > 0.018 as the criteria for selection.

#### 3.1.4 Drug-target-disease-pathway network

**Fig. 5:**
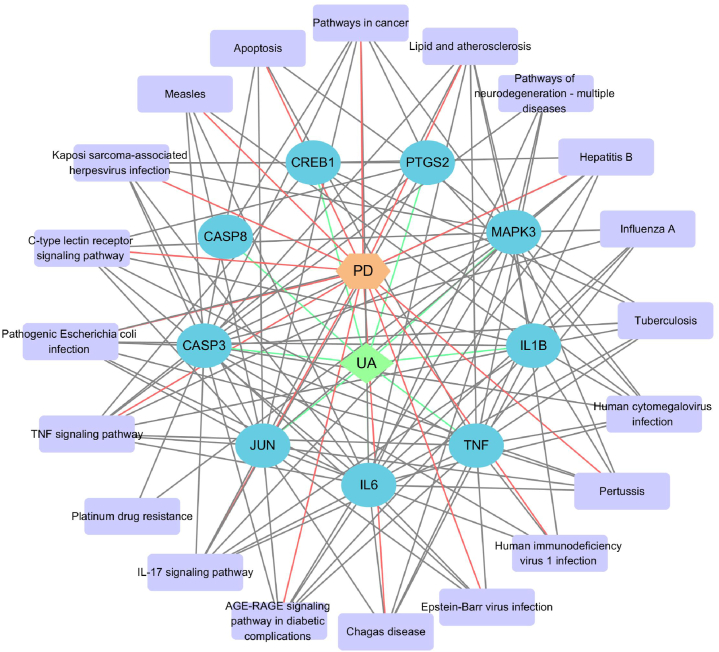
Relationship network between potential therapeutic targets against UA and PD.

An interactive network for the treatment of PD using UA was established. The network was represented using various color and shape patterns. Fig. 5 displays the UA-target-PD-pathway network, consisting of 31 nodes and 233 edges. In this network, the green rhombus represents UA, the saffron yellow polygon represents PD, the blue oval represents the common target genes of UA and PD, and the purple box represents the pathway of these targets. These findings suggest that UA may exert regulatory control over PD by targeting multiple genes.

### 3.2 Molecular docking

**Fig. 6:**
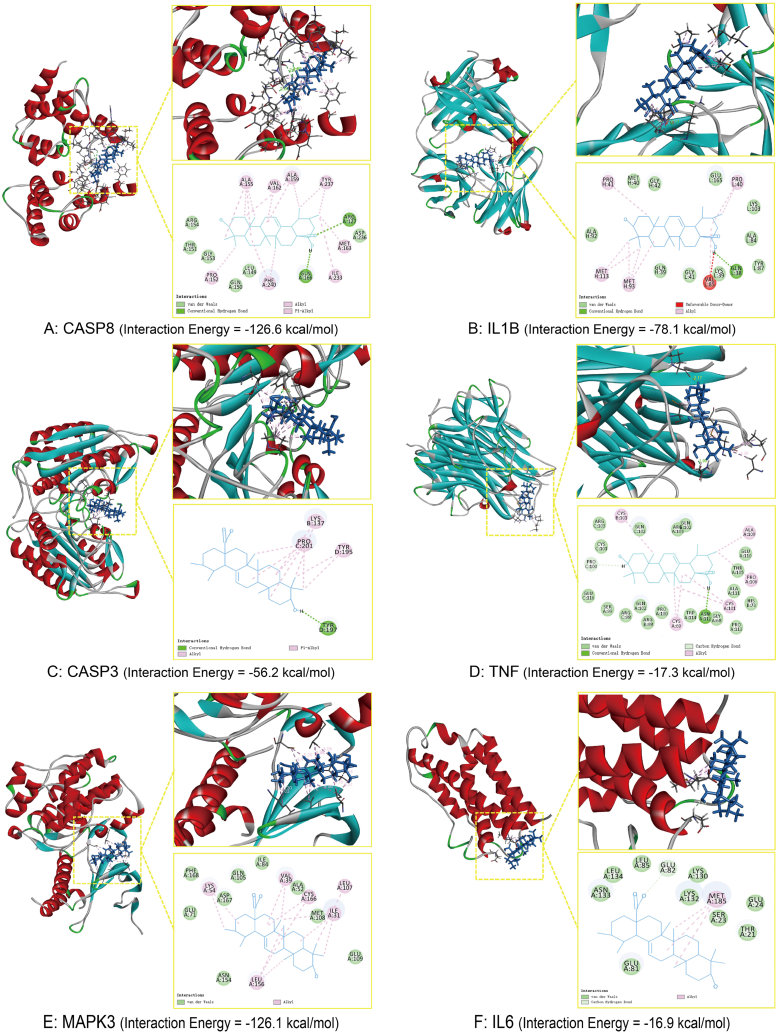
Molecular docking results of UA and the corresponding protein of the gene targets.

To further investigate the potential binding patterns and reliability of interactions between UA and its potential targets, molecular docking was employed. Previous research has demonstrated that a decrease in binding energy between the small molecule compound and the ligand leads to a more stable conformation, thereby increasing the likelihood of their interaction. Notably, when the binding energy falls below −7 kJ/mol, the conformation exhibits strong binding activity [24]. In this particular investigation, the binding energy of 6 core targets associated with UA and PD was found to be lower than −7 kJ/mol. Furthermore, UA engages with these targets through various mechanisms, including Hydrogen Bond, van der Waals, Alkyl, Conventional Pi-Alkyl and so on, indicating that the compounds and these receptors have a certain binding activity (Fig. 6A–F). According to binding energy and the number of chemical bonds, 6 core targets were finally selected: CASP8, IL1B, CASP3, TNF, MAPK3 and IL6.

Molecular dynamics (MD) simulation has been extensively utilized to evaluate the structural characteristics of protein-ligand systems and investigate the binding stability between proteins and molecules [25]. To further understand the differences in the binding processes of the six target proteins with ursolic acid, we performed 100ns MD simulations using the Schrödinger suite version 2021 (Schrödinger LLC, New York, NY) software. During this process, the root-mean-square deviation (RMSD) was adopted as an indicator to reflect the fluctuation process of the complex. Higher RMSD values and corresponding fluctuations in the complex indicate more intense movements between the protein receptor and small molecule ligand and suggest poor binding stability.

This analysis provides valuable insights into understanding the interactions between different target proteins and UA. Within the 0–100 ns range, the RMSD values of Supplemental Fig. 1 A-C (provided in the Suppl. Fig. 1 docx) consistently remained stable, indicating highly stable binding interactions for the complexes of UA-MAPK3, UA-CASP3, and UA-CASP8. Additionally, as shown in Supplemental Fig. 1 D, the RMSD values exhibited significant fluctuations within the 0–35 ns range and subsequently stabilized between 35 and 100 ns, suggesting a relatively stable binding of UA with IL1B. In contrast, the results of Supplemental Fig. 1 E and F demonstrate continuous RMSD fluctuations throughout the 0–100 ns range, suggesting that the UA-IL6 and UA-TNF complexes have unstable binding interactions.

**Fig. 7:**
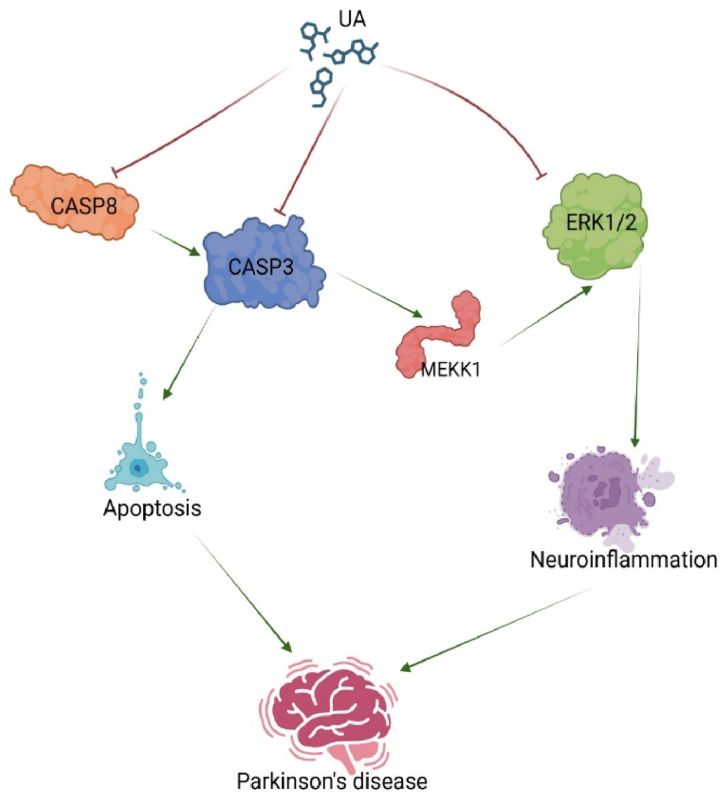
Hypothetical pathway diagram for UA treatment of PD.

Notably, studies have shown that caspase-3 triggers the activation of ERK1/2 via a mechanism that is independent of protease activity and reliant on ceramide signaling, in this process, MEKK1 kinase is a substrate for caspase-3. TNF-α and IL-1β activate caspase-3, triggering a cascade of downstream signaling events and the transcription of inflammatory genes, culminating in the release of cytokines, including NF-κB, IL-6, and nitric oxide (NO). Combining the results of molecular docking and dynamics simulation, we propose a dual mechanism by which UA may exert its therapeutic effects in PD. As illustrated in Fig. 7: firstly, UA could mitigate neuroinflammation and enhance PD symptomatology by inhibiting the activation of extracellular signal-regulated kinase 1/2 (ERK1/2), thus attenuating ERK1/2-mediated inflammatory pathways. Secondly, UA may impede the progression of PD by suppressing the expression of CASP8 and CASP3, effectively preventing neuronal apoptosis.

### 3.3 Experimental verification

#### 3.3.1 Effects of UA on motor dysfunction in PD mice

**Fig. 8:**
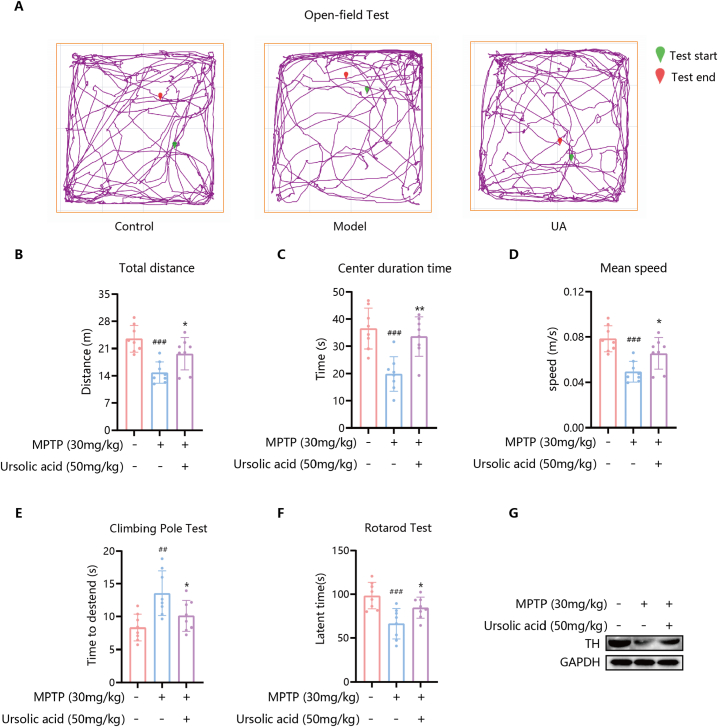
UA ameliorates motor dysfunction in PD mice. A–D: The movement track of mice in Open-field Test (A), Total Distance (B), Center duration time (C), Mean speed (D) of mice movement detected by Open-field Test, n = 8; E: Time of mice to descend to the bottom of the pole detected by Climbing Pole Test, n = 8; F: Time from start to fall detected by rotarod test, n = 8; G: Representative protein bands of TH and GAPDH. ^##^P＜0.01, ^###^P＜0.001, compared with Control group; *P＜0.05, **P＜0.01, compared with Model group.

The results of the Open-field Test (Fig. 8A–D) showed that, compared to the Control group, mice in the Model group exhibited a significant decrease in Distance, Center duration time, and Mean speed (P < 0.001) in the open-field apparatus. In contrast, mice in the UA group showed a significant increase in Distance (P < 0.05), Center duration time (P < 0.01), and Mean speed (P < 0.05) compared to the Model group. In the Climbing Pole Test (Fig. 8E), the Model group mice took significantly longer to climb to the bottom compared to the Control group (P < 0.01). However, the UA group mice demonstrated a significant reduction in the time taken to climb to the bottom compared to the Model group (P < 0.05). The results of the Rotarod Test (Fig. 8F) indicated that mice treated with MPTP showed worse performance in rotarod performance compared to normal controls (P < 0.001). The UA group exhibited improvement in rotarod performance compared to the Model group (P < 0.05). These results collectively suggest that mice with PD induced by MPTP experience motor dysfunction, which can be ameliorated by UA treatment.

#### 3.3.2 Effect of UA on the expression of TH protein

The amelioration of the loss of TH-positive dopamine neurons was confirmed by Western blot analysis (Fig. 8G), the MPTP produced a remarkable reduction in TH expression compared with the Control group. UA significantly ameliorated the loss of TH expression in PD mice induced by MPTP.

#### 3.3.3 Effect of UA on SH-SY5Y cell viability

**Fig. 9:**
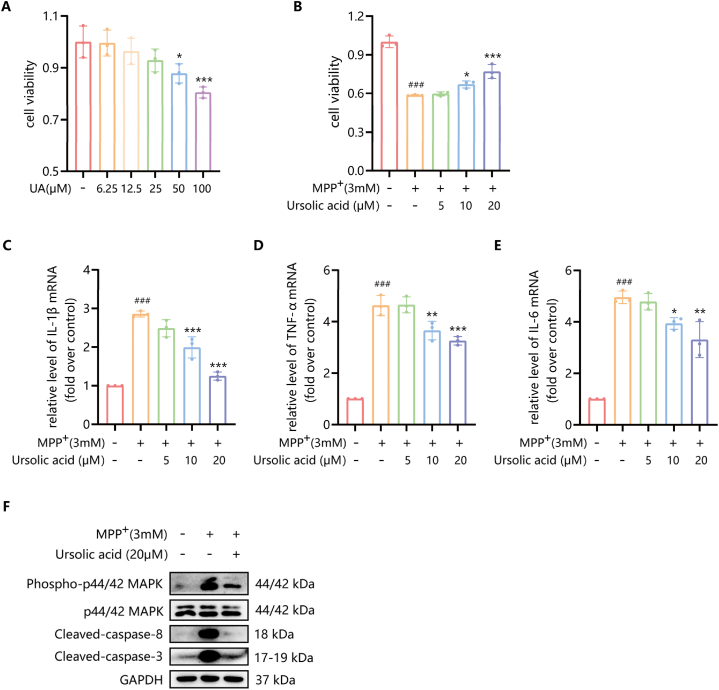
CCK-8 assay showed the cytotoxic effects of UA (0–100 μM) on SH-SY5Y cells (A) and the therapeutic effect of UA (5–20 μM) on MPP^+^-induced SH-SY5Y cells (B). RT-qPCR showed the relative expression levels of inflammatory cytokines after SH-SY5Y was treated with several concentrations of UA (5–20 μM), with or without 3 mM MPP^+^ (C–E). Western blot analyses showed the effect of UA (20 μM) on the expression levels of CASP8, CASP3, and MAPK3 on MPP^+^-induced SH-SY5Y cells (F). The results are shown as the mean ± SEM. ^###^P < 0.001 compared with the control group, *P < 0.05, **P < 0.01, ***P < 0.001 compared with the MPP ^+^ -induced group (n = 3).

The cytotoxic effects of UA were assessed by the CCK-8 assay. SH-SY5Y cells were treated with UA at various concentrations ranging from 0 to 100 μM (Fig. 9A). This analysis revealed that with increasing of drug concentration, the cell viability gradually decreased, and UA did not possess notable toxicity to the cells at concentrations of 0–25 μM (Fig. 9A). Then, we induced SH-SY5Y cells with 3 mM MPP^+^ for 24 h to simulate the damage of neurons in PD, and before that several concentrations of UA (5–20 μM) were added to cells for pre-protection. As shown in Fig. 9B, compared with the normal group, the cell viability was significantly reduced after 3 mM MPP^+^ induction (P < 0.001), while the damage was mitigated by UA at 10 μM and 20 μM (P < 0.05, P < 0.001).

#### 3.3.4 Effect of UA on the expression of inflammatory cytokines

RT-qPCR was used to examine the relative expression levels of inflammatory cytokines after SH-SY5Y was treated with several concentrations of UA (5–20 μM), with or without 3 mM MPP^+^.

The results showed that compared with the control group, the gene expression of IL-1β, TNF-α, and IL-6 in cells induced by MPP^+^ was significantly increased (P < 0.001) (Fig. 9C–E). When 10 μM and 20 μM UA were added to the cells induced by MPP^+^, the gene expression of IL-1β, TNF-α, and IL-6 gradually decreased with increasing concentration compared with the model group. These results suggest that UA may improve the MPP^+^-induced PD model by downregulating the gene expression of inflammatory factors such as IL-1β, TNF-α, and IL-6.

#### 3.3.5 Effect of UA on the expression of CASP8, CASP3 and MAPK3 proteins in SH-SY5Y cells

To analyze the effect of UA on protein expression of potential targets associated with PD, CASP8, CASP3, and MAPK3 expression levels were determined through Western blot analysis. The results revealed that, compared with the control group, phosphorylated p44/42 MAPK protein expression increased in the model group, while 20 μM UA inhibited the phosphorylation of p44/42 in MPP^+^-stimulated SH-SY5Y cells (Fig. 9F). In addition, the level of cleaved-caspase-8 and cleaved-caspase-3 protein in the model group was increased, while decreased after UA treatment (Fig. 9F). These results suggest that UA may improve the MPP^+^-induced PD model by inhibiting the maturity of caspase-8 and caspase-3, and the phosphorylation of p44/42 MAPK protein.

## 4 Discussion

Ursolic acid exhibits a wide range of biological activities, such as anti-inflammatory [26], antioxidant [27], anti-anxiety [28], anti-depressant [29], and neuroprotective pharmacological effects [30]. Parkinson's disease is a neurodegenerative disease, and recently, the link between neuroinflammation and PD has received rising attention. More and more evidence has suggested that central and peripheral inflammation plays a crucial role in the pathological features and symptoms of PD [31]. The latest research shows that α-synuclein, a pathogenic protein in PD, is closely related to neuroinflammation caused by neurodegeneration. Generally, aggregated α-synuclein can be released from damaged neurons and recognized by Toll-like receptors on microglia, activating the NF-kB pathway and the NLRP3 inflammasome to induce neuroinflammation [32].

In this study, 147 UA action targets and 790 PD-related targets were screened through network pharmacology, and 35 potential UA anti-PD targets were obtained after intersection. Next, we performed GO and KEGG pathway enrichment analysis on these 35 targets. These two analysis results identified the regulatory role of the common 35 targets of UA and PD in biological functions and signaling pathways.

GO analysis revealed that the aforementioned overlapping genes primarily affect biological processes such as apoptotic process and neuron apoptotic process. The KEGG enrichment analysis shows that the targets of UA in exerting its anti-PD effect are primarily enriched in Apoptosis signaling pathway, Pathways of neurodegeneration-multiple diseases, TNF signaling pathway, and so on. The above research indicates the potential therapeutic value of UA in the treatment of PD, and it is closely related to the Apoptosis signaling pathway and its targets.

We then constructed a drug-target-disease-pathway network to reveal the possible molecular mechanism of UA in treating PD. Prediction of key targets for the treatment of PD with UA through protein interaction networks PPI network analysis revealed that UA may play an anti-PD role by regulating 9 target proteins such as IL1B, TNF, and CASP3. These genes are directly or indirectly involved in cell inflammation, apoptosis, pyroptosis, oxidative stress, and other processes.

To explore the binding ability of the 9 key targets to UA, we performed molecular docking and molecular dynamic simulation of these targets to UA. The molecular docking results demonstrated that UA exhibits favorable binding affinity with the 6 core genes (CASP8, IL1B, CASP3, TNF, MAPK3, and IL6). After integrating the results of molecular docking and molecular dynamics simulations, we realized that UA has strong binding capabilities with the three proteins MAPK3, CASP8, and CASP3.Furthermore, by consulting the literature, we found that the aforementioned three target genes are closely related to apoptosis and neuroinflammation [33,34].

Caspase-8 represents the molecular switch that controls apoptosis, necroptosis and pyroptosis [35]. Studies have shown that CASP8 can induce the activation of ASC, thereby activating caspase-1and inducing pyroptosis [36]. The activation of caspase-8 and caspase-3 are the agents of apoptosis and regulate the activation of microglia. Deletion of caspase-8 blocks microglial proinflammatory activation and confers protection in a MPTP neurodegeneration model [37]. Caspase-3 is an effector of apoptosis in experimental models of PD [38]. The present study showed that rosinidin inhibits caspase-3 expression and restores neurotransmitter levels in rotenone-activated PD [39]. Our results also showed that UA significantly decreased the levels of cleaved caspase-8 and caspase-3.

MAPKs are serine/threonine protein kinases that promote the diversity of cellular functions across multiple cell types. There are three main types of the MAPK subfamily: theERK1/2, c-JunNH2-terminal kinases (JNK), and p38 kinases [40]. A growing body of research suggests that ERK1/2 has a death-promoting effect in both in vitro and in vivo neuronal death models [41]. An in vivo experiment confirmed that nootkatone can inhibit the expression of MAPK3 by activating the PI3K/Akt signaling pathway, reducing neuroinflammation, and ultimately improving the symptoms of rotenone-induced PD [42]. Our study also found that UA can reduce the expression of MAPK3 protein in PD cell models.

There are many causes of idiopathic PD, and some studies have suggested that inflammation, microglia and inflammatory bodies play a key role in the pathogenesis of PD [43]. In PD, microglia become highly reactive, producing various neuroinflammatory molecules, such as IL-1β, IL-18, IL-6, TNF-α and chemokines, which are toxic to tissue and can damage neurons [44]. Our experimental results from animal behavior studies indicate that UA has a beneficial effect on motor dysfunction in PD model mice. Subsequent in vitro experiments showed that UA can reduce the gene expression of IL-1β, IL-6, and TNF-α in the MPP^+^-induced PD cell model.

Based on this foundation, we speculate that UA may exert its anti-PD effects by regulating the apoptotic signaling pathway and neuroinflammatory signaling pathway, which may be realized through the regulation of the above 3 target proteins (MAPK3, CASP8, and CASP3). To verify this conjecture, we further conducted in vitro experiments. We established an MPP^+^-induced SH-SY5Y cell model, and the expression of TNF-α, IL-6, IL-1, CASP8, CASP3 and MAPK3 was assessed. The results showed that UA decreased the gene expression levels of TNF-α, IL-6 and IL-1 and inhibited the protein expression of MAPK3, CASP8 and CASP3. The results of this study indicate that UA can ameliorate PD, and its molecular mechanism may involve the inhibition of apoptosis and neuroinflammation in nerve cells by targeting MAPK3, CASP8 and CASP3. At present, there are several studies on the pharmacokinetics of UA, including both intravenous and intragastric administration in mice and rats, as reviewed by Jinhua W [45]. Based on these studies, UA is classified as a class IV drug in the biopharmaceutical classification system due to issues such as low water solubility and bioavailability. Nevertheless, UA is one of the most common active components in plants and demonstrates a wide range of biological activities, leading to a surge in research on its suitable dosage forms. For instance, one study has shown that the development of a new type of hybrid PLGA/lipid nanoparticle of UA may help exert therapeutic activity against pancreatic ductal adenocarcinoma [46]. Additionally, other research has led to the development of hyaluronic acid/dextran-based polymeric micelles that deliver UA to help treat cancer multidrug resistance [47]. Furthermore, studies have indicated that UA derivatives may exhibit improved absorption and enhanced biological activity [48], thus holding greater potential for clinical applications.

## 5 Conclusions

In summary, this study systematically analyzed the potential mechanism of action of UA in the treatment of PD through network pharmacology technology and initially verified the target of action by molecular docking technology. Subsequently, the impact of UA on PD was assessed through both in vivo and in vitro experiments. The main conclusion that can be drawn is that UA can treat PD through multiple targets in the apoptosis and neuroinflammation signaling pathway. Nonetheless, this study still has some limitations. In a follow-up study, we will continue to conduct in vivo and in vitro experiments to further clarify the mechanism of action of UA in the treatment of PD.

**Table:** Abbreviations

ERK1/2	extracellular signal-regulated kinases 1 and 2
FBS	fetal bovine serum
GO	Gene Ontology
JNK	c-JunNH2-terminal kinases
KEGG	Kyoto Encyclopedia of Genes and Genomes
MD	Molecular dynamics
MPP+	N-Methyl-4-phenylpyridinium iodide
NO	Nitric Oxide
PD	Parkinson's disease
PPI	protein-protein interaction
PT	pentacyclic triterpenoid
RMSD	root-mean-square deviation
RT-qPCR	Real-time quantitative PCR
TCMSP	Traditional Chinese Medicine Systems Pharmacology
TH	tyrosine hydroxylase
UA	Ursolic acid
α-syn	alpha-synuclein

## Ethics statement

The work described has been carried out in accordance with either the U.K. Animals (Scientific Procedures) Act, 1986 and associated guidelines, the European Communities Council Directive 2010/63/EU or the National Institutes of Health – Office of Laboratory Animal Welfare policies and laws. All animal studies comply with the ARRIVE guidelines. This study was reviewed and approved by the Ethics Committee of Jiangsu Vocational College of Medicine with the approval number: XMLL-2023-710, dated 2023.02.21. The Animal Ethics Committee of The First People's Hospital of Yancheng approved the animal work (SYXK 2023-0005).

## Data availability statement

The data associated with the study not been deposited yet into a publicly available repository. If you have any needs, please contact the corresponding author by email to obtain more information about the data.

## CRediT authorship contribution statement

**Ao Sun:** Writing – original draft, Investigation, Conceptualization. **Yu-fei Li:** Writing – review & editing, Writing – original draft, Methodology, Conceptualization. **Yang Miao:** Supervision, Project administration. **Hong-xia Wang:** Resources, Project administration. **Lin-lin Zhang:** Resources, Funding acquisition.

## Declaration of competing interest

The authors declare that they have no known competing financial interests or personal relationships that could have appeared to influence the work reported in this paper.
