# Supplementary material for: Research on the mechanism of Ursolic acid for treating Parkinson's disease by network pharmacology and experimental verification
Source: Heliyon. 2024 Jul 8;10(14):e34113. doi: 10.1016/j.heliyon.2024.e34113 (PMC11301175; doi:10.1016/j.heliyon.2024.e34113)
Supplement: Multimedia component 1 [file mmc1.docx]

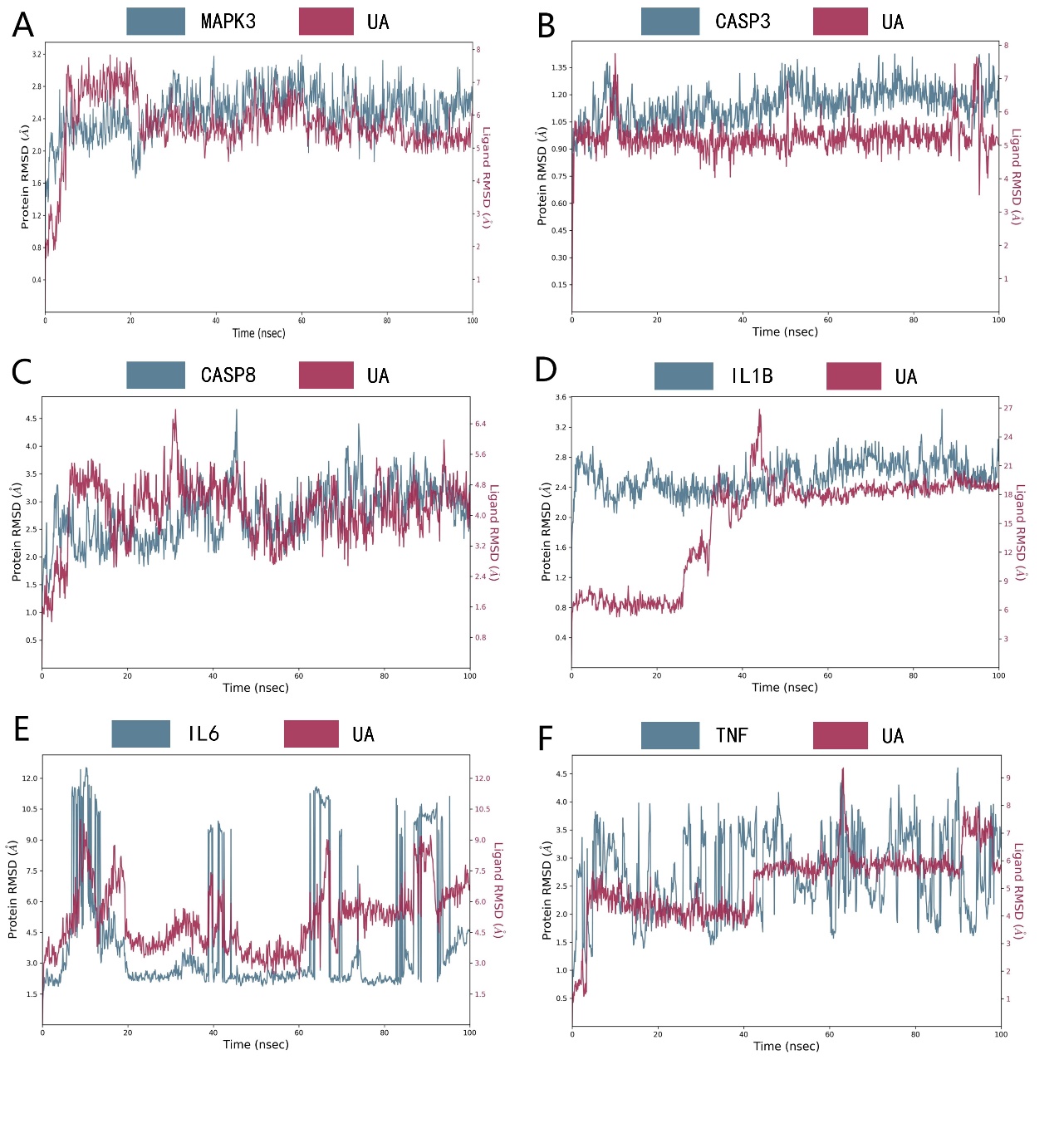


**Supplemental Figure 1. Molecular dynamics simulation analysis of UA with 6 target proteins.** During the simulations, we ensured that the protein-ligand complexes were solvated in a physiologically relevant environment and subjected to a series of energy minimizations and equilibration steps to achieve stable starting conditions. The production runs were carried out for an extended period to capture the dynamic behavior of the systems. After that, we successfully generated the RMSD (Root Mean Square Deviation) plots for both the ligand and the proteins involved in our study. The fluctuations in the RMSD values of the protein’s main chain Cα atoms and UA are presented, reflecting the deviation of the Cα from its initial position within the complex system. The RMSD plot of the Cα atoms for the complexes of (A) UA with MAPK3, (B) UA with CASP3, (C) UA with CASP8, (D) UA with IL1B, (E) UA with IL6, (F) UA with TNF.
