# Supplementary material for: Research on the mechanism of Ursolic acid for treating Parkinson's disease by network pharmacology and experimental verification
Source: Heliyon. 2024 Jul 8;10(14):e34113. doi: 10.1016/j.heliyon.2024.e34113 (PMC11301175; doi:10.1016/j.heliyon.2024.e34113)
Supplement: Multimedia component 2 [file mmc2.docx]

The Original Images of Western Blots


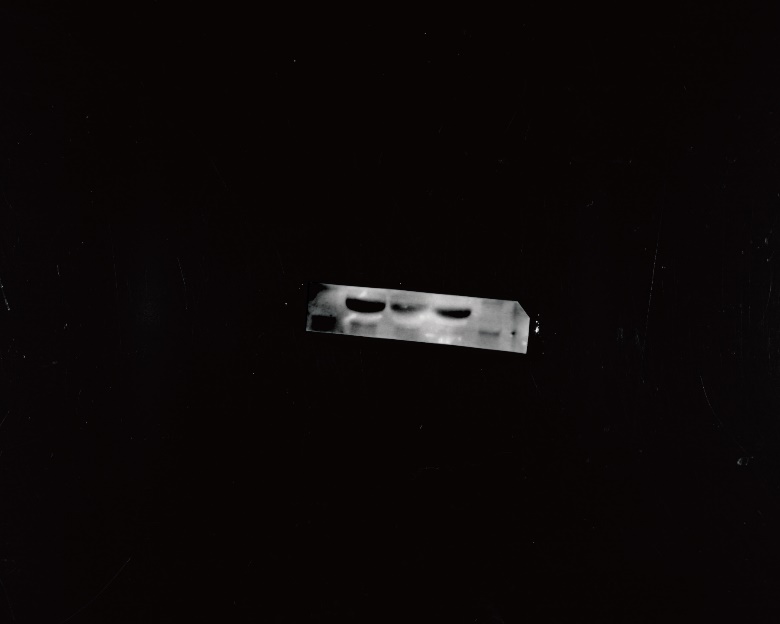

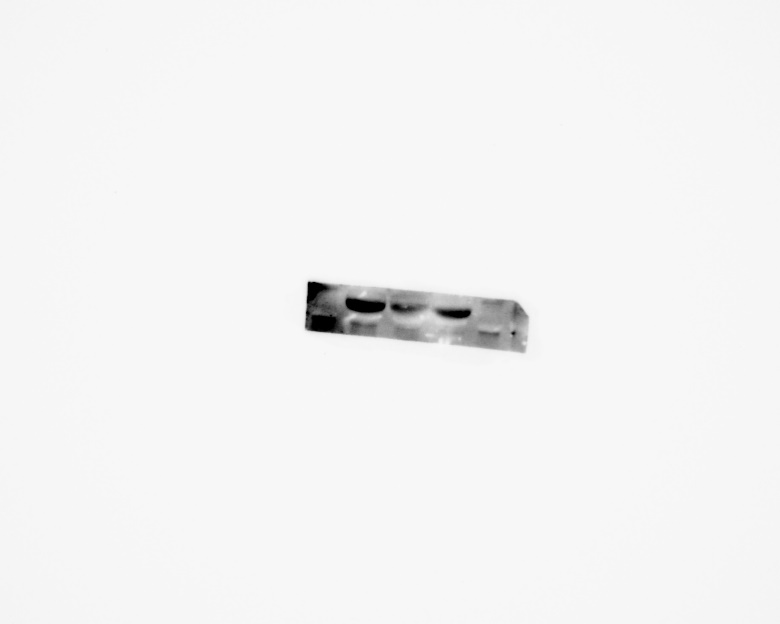


**Figure 8. (G)** Immunoblots of Tyrosine Hydroxylase (TH)


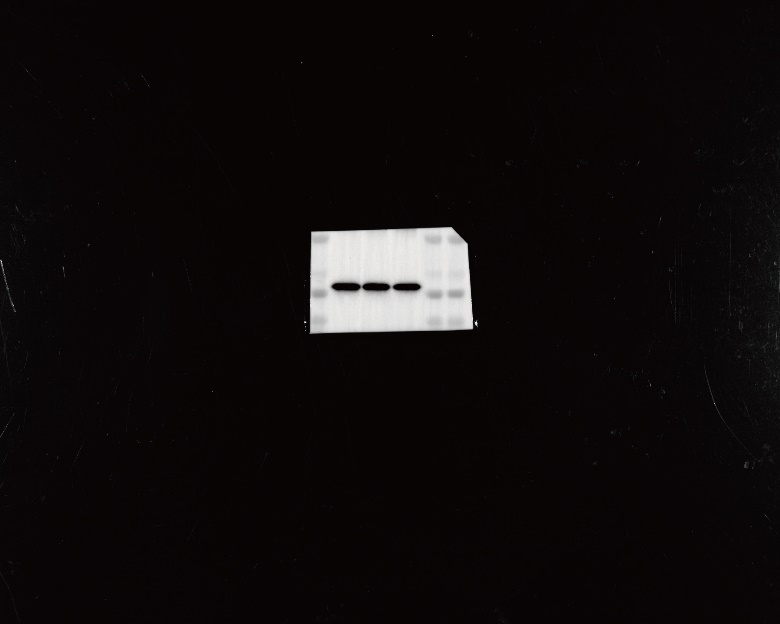

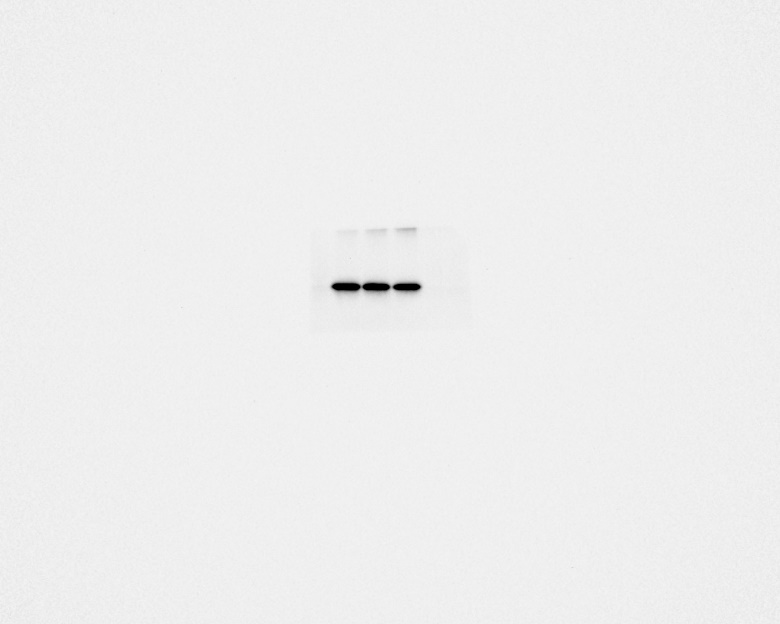


**Figure 8. (G)** Immunoblots of GAPDH


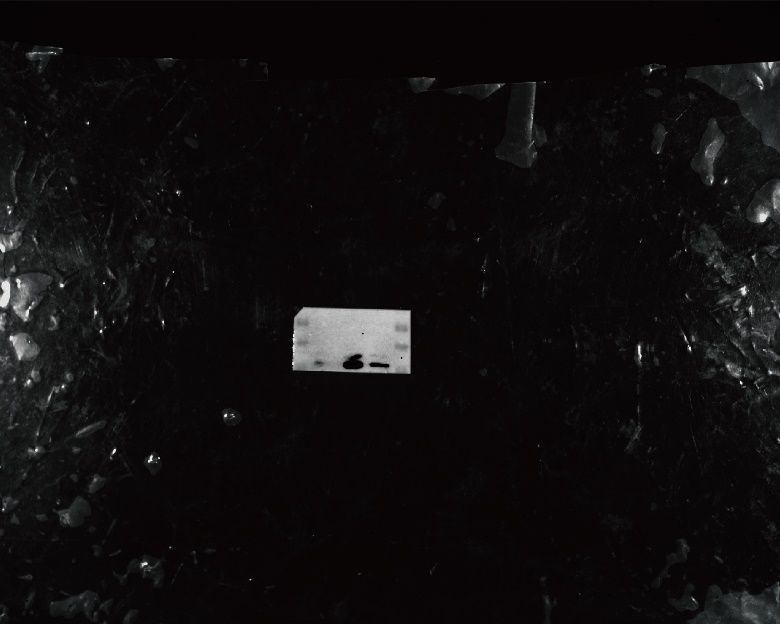

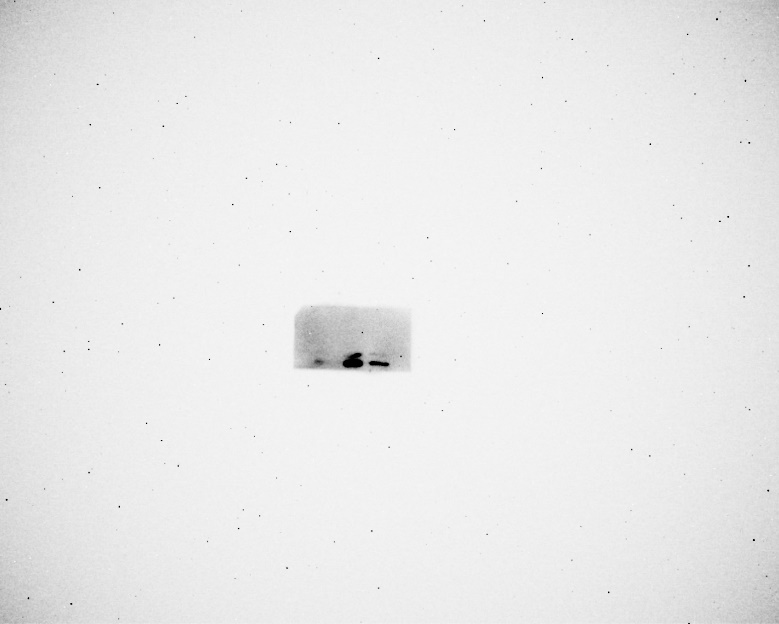


**Figure 9. (F)** Immunoblots of Phospho-p44/42 MAPK


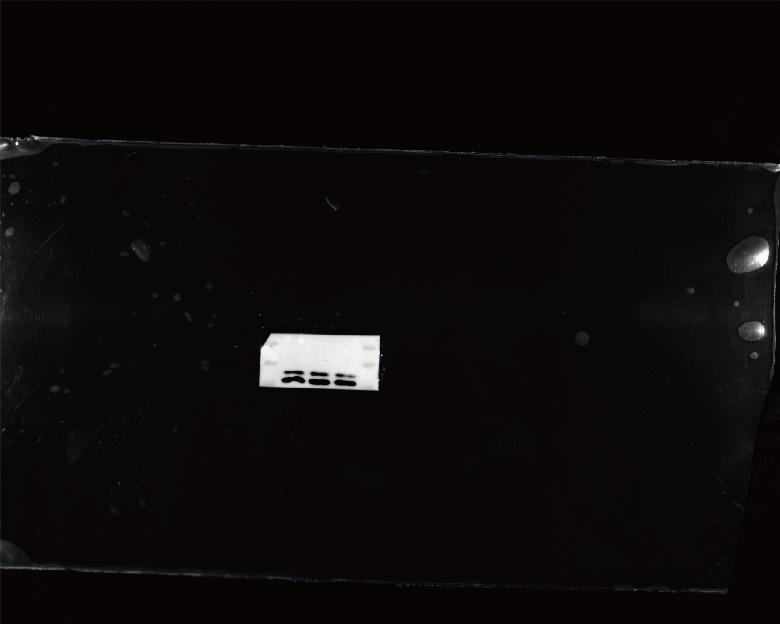

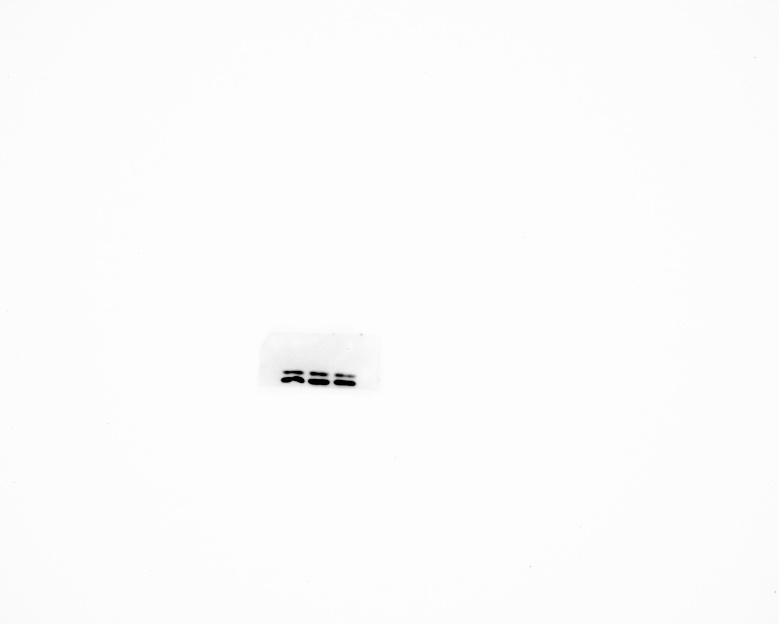


**Figure 9. (F)** Immunoblots of p44/42 MAPK


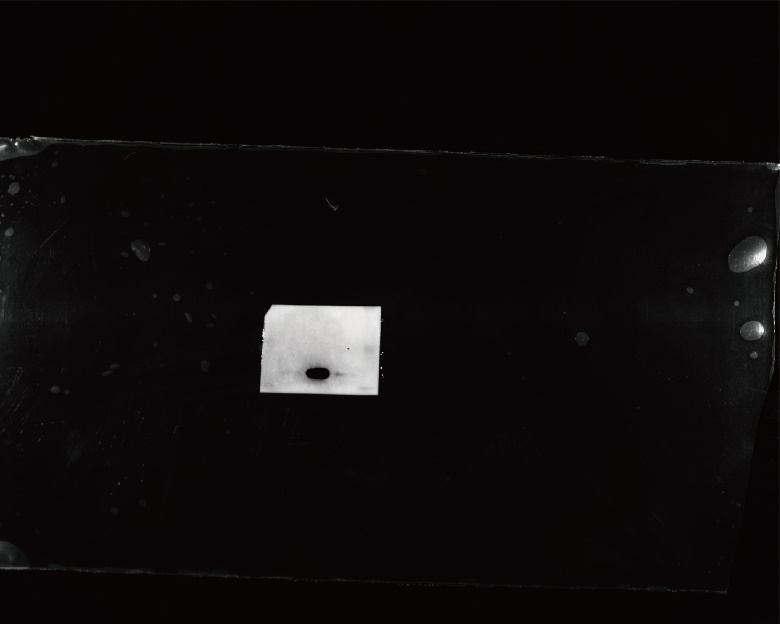

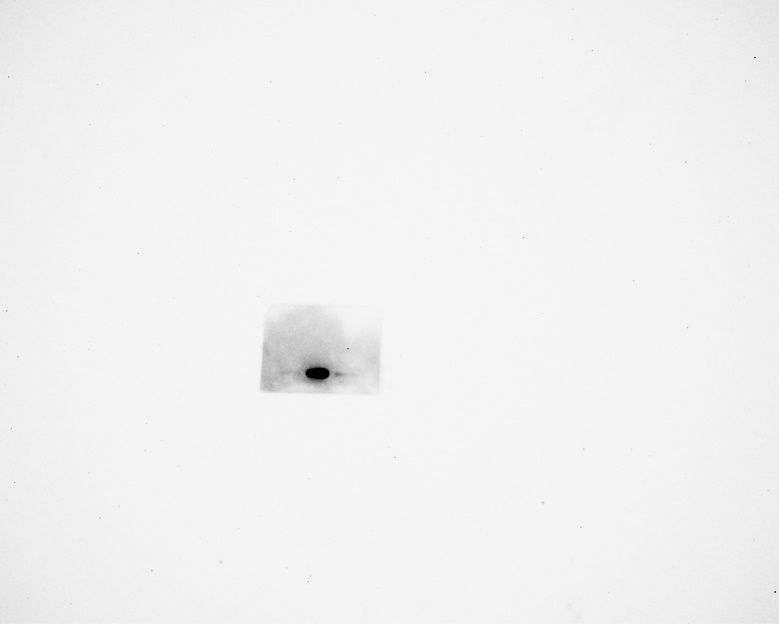


**Figure 9. (F)** Immunoblots of Cleaved-Caspase-8


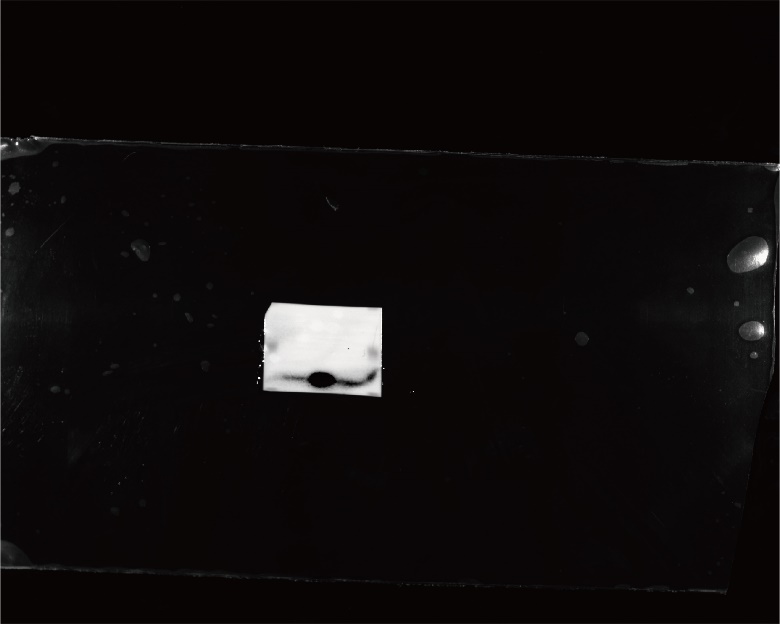

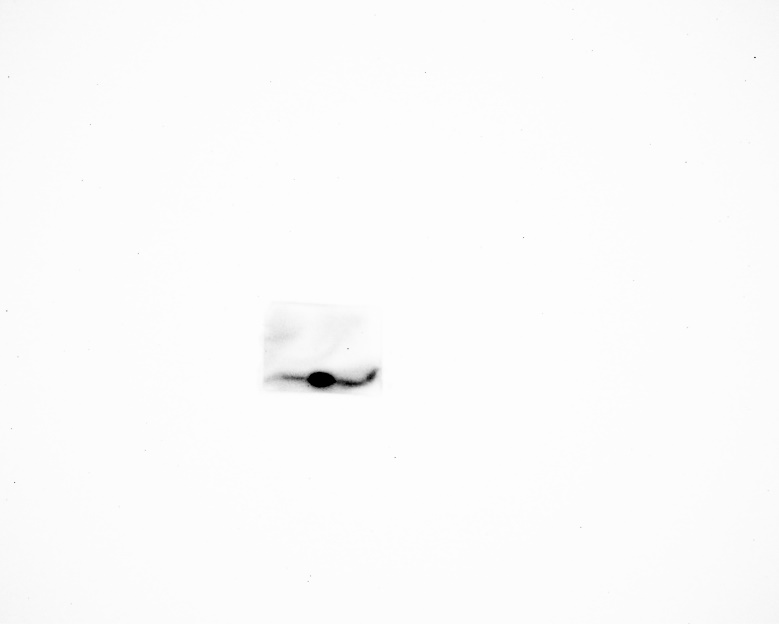


**Figure 9. (F)** Immunoblots of Cleaved-Caspase-3


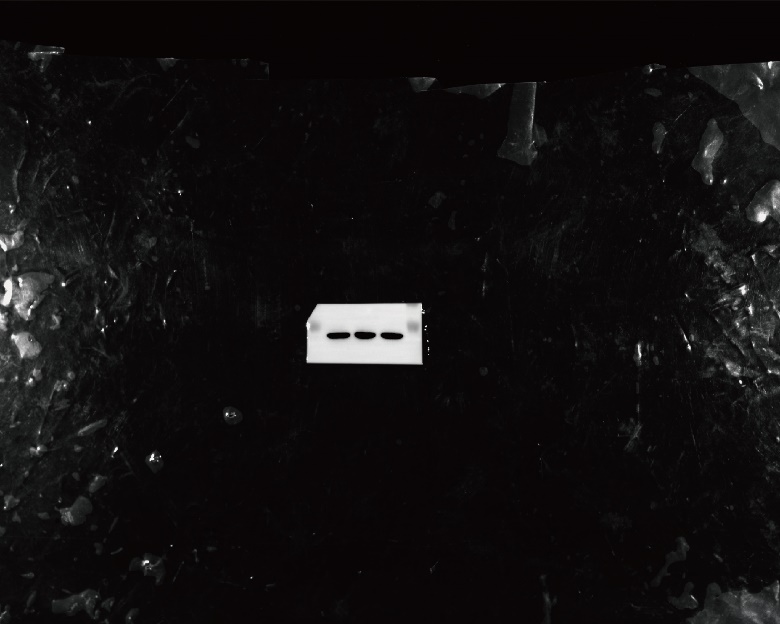

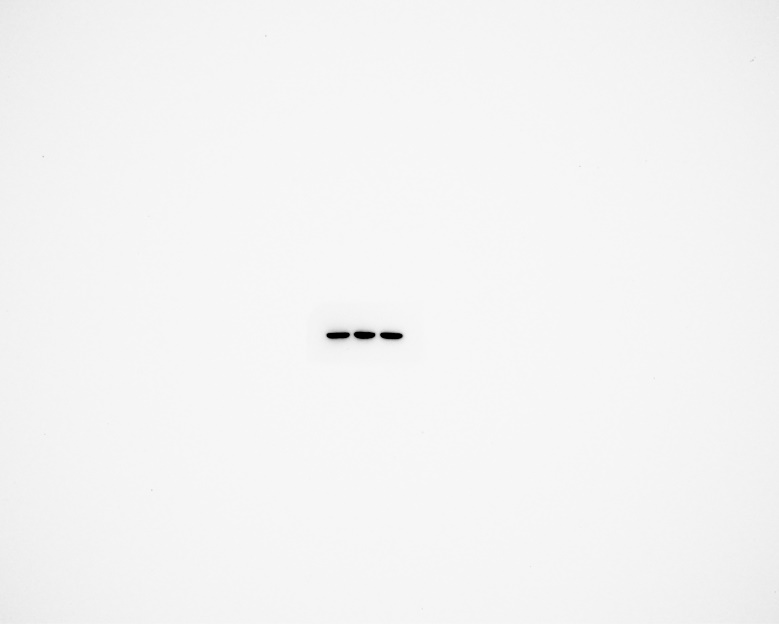


**Figure 9. (F)** Immunoblots of GAPDH
